# Preliminary study of perceived cardiovascular disease risk and risk status of adults in small rural and urban locations in Ibadan, Nigeria

**DOI:** 10.3934/publichealth.2023015

**Published:** 2023-03-14

**Authors:** Nse A Odunaiya, Opeyemi M Adegoke, Abiodun M Adeoye, Oluwafemi O Oguntibeju

**Affiliations:** 1 Department of Physiotherapy, Faculty of Clinical Sciences, College of Medicine, University of Ibadan, Ibadan, Nigeria; 2 Department of Medicine, University of Ibadan/University College Hospital, Ibadan, Nigeria; 3 Department of Biomedical Sciences, Faculty of Health & Wellness Sciences, Cape Peninsula University of Technology, Bellville Campus 7535, Cape Town, South Africa

**Keywords:** adults, cardiovascular disease, Nigeria, perceived cardiovascular disease risk, risk status, rural, urban

## Abstract

The burden of cardiovascular disease (CVD) has been on the rise in developing countries like Nigeria recently. Studies on perceived CVD risk and the risk status of adults in Ibadan are not readily available, hence this study. A mixed-method design involving a cross-sectional survey and an exploratory qualitative study was utilized. Convenience sampling was used to recruit 418 participants (209 from rural and 209 from urban) for the cross-sectional survey, while purposive sampling was used to recruit 14 participants for the qualitative aspect. The INTERHEART risk score and the Perception of Risk of Heart Disease Scale were used to investigate participants' CVD risk status and perceived risk, respectively. The data from the cross-sectional survey were summarized by using descriptive statistics, and the data were then analyzed by using the chi-square test of association and a multiple logistic regression model, while content thematic analysis was used to analyze the qualitative data. In the rural and urban areas, respectively, 39.7% and 52.2% had a positive perception of CVD risk. In the rural and urban areas, 44% and 41.6% of individuals respectively had moderate-to-high risk of CVD. Participants with at least secondary school education [2.66 (0.61–11.53)] and participants in the urban area [2.62 (0.78–7.08)] had twice higher odds of positive CVD risk perception. Males [3.91 (1.58–9.68)], adults aged 40 and above [1.59 (0.63–4.00)] and urban dwellers [1.21 (0.33–4.39)] had higher odds of a high CVD risk status. The qualitative aspect of the study corroborated the findings from the survey, as many participants did not perceive themselves as being at risk of CVD. The majority of the participants in this study were found to have a moderate-to-high risk of CVD, and many had a negative perception of their risk. Health education and CVD prevention programs are required to curb the burden of CVD.

## 1. Introduction

Cardiovascular disease (CVD) is a global epidemic and a major public health crisis [1]. According to a World Health Organization report [2], 17.9 million people died from CVD in 2019, accounting for 32% of world mortality that year. CVD is the leading non-communicable disease in sub-Saharan Africa, accounting for more than 20% of all fatalities and 7% of disability-adjusted life years [3],[4]. CVD has become more prevalent in Nigeria recently [5], owing to increased urbanization and a westernized lifestyle [6],[7]. CVD accounts for 11% of all deaths in Nigeria [8]. The rise in CVD burden is generally thought to be the result of a complex interaction among a conglomeration of risk factors such as an unhealthy diet, a low physical activity level, tobacco smoking, socioeconomic stressors and financial constraints at both the national and personal levels [6],[9]. According to the World Health Organization, the burden of CVD will continue to rise, and, by 2030, the burden of CVD would have surpassed that of infectious diseases in developing countries [10].

Estimating CVD risk status using laboratory-based or non-laboratory-based approaches is a crucial step toward lowering the burden of CVD [11]. Due to the high cost of laboratory investigations, the use of non-laboratory-based risk algorithms, such as the INTERHEART risk score, has been promoted in low-resource settings [12]. Following CVD risk estimation, individuals are classified as having a low, moderate or high risk of CVD based on their risk factors [13]. CVD risk estimation impels the early detection of CVD risk and underpins the need for lifestyle modification and the management of risk factors [14]. Previous studies in Nigeria have reported the high prevalence and clustering of CVD risk factors among adults and adolescents [15]–[20], but studies on the CVD risk status of adults, particularly those living in rural locations, are not common. Studies investigating the perceived risk of CVD are equally sparse in Nigeria. To the best of our knowledge, this is the first study investigating both perceived CVD risk and CVD risk status among Nigerian adults residing in urban as well as rural locations in a single study.

An exploration of perceived CVD risk in a target population is crucial, given the fact that risk perception significantly predicts health behavior [21]. Hochbaum et al. [22] proposed the health belief model, which suggests that an individual's perception of disease susceptibility and severity can impact health behavior. Therefore, proper perception of CVD risk is a prerequisite for lifestyle modification and treatment adherence [23]. Lack of accurate perceived CVD risk has the potential to impede the adoption of early preventive behavior or the pursuit of early intervention, even in the presence of symptoms. Since several factors such as culture, educational attainment, socioeconomic status and religious beliefs can influence lifestyle habits and health behavior in different ways, it becomes important to investigate the perceived CVD risk and risk status of adults in rural and urban locations. In this paper, we report the perceived CVD risk and risk status of adults in selected rural and urban locations in Ibadan, Nigeria, as well as the factors associated with them.

## 2. Materials and methods

### 2.1. Study design and study population

This study utilized a mixed methods design, involving a cross-sectional survey and an exploratory qualitative component (focus group discussion (FGD)). The research was conducted in Ikereku, a small town in Akinyele Local Government Area, which served as the rural location, and in Bodija/Agodi, in the Ibadan North Local Government Area, which served as the urban location. The rural area has a low population density, with peasant farming and trading being the main occupation of the people. Alternatively, the urban area is densely populated and has various official and residential structures, and the majority of the people are either government-employed or private business owners. The people in the two locations are generally of the Yoruba ethnicity of Nigeria. Adults aged 20 and above who could comprehend and converse in English and/or Yoruba (the local language spoken in study locations) and who had no history of diagnosed CVD were eligible to participate in the study. Pregnant women were excluded from the study since their abdominal sizes compromise waist circumference measurement.

### 2.2. Ethical approval of research

The University of Ibadan/University College Hospital Health Research Ethics Committee gave ethical approval for this study, with reference number UI/EC/21/0268. Relevant authorities in the locations where the study was conducted also gave permission. Participants signed an informed consent form after the rationale and procedure of the study were explained to them, with an assurance of confidentiality and anonymity. Some participants in the rural location who were not literate gave verbal consent.

### 2.3. Sampling technique and sample size determination

A sample of convenience was used for the cross-sectional survey, while participants for the exploratory qualitative component of the study (FGD) were recruited by applying a purposive sampling technique. The sample size method for estimation of prevalence [24] was used to determine the minimum number of participants for the cross-sectional survey in this study:

n=z2(1−α2)pqL2 (1)

In Nigeria, there appears to be no national data on the prevalence of CVD. As a result, the CVD mortality rate was used to determine the sample size in this study. According to the World Health Organization [8], the estimated rate of CVD mortality in Nigeria was 11%. There appears to be no current estimated rate, however, since the prevalence of CVD is expected to increase, 14% was taken as the rate for the year 2021, when the study was conducted.

$$\begin{array}{c} n=\frac{1.96^{2}\times 0.14\times 0.86}{0.05^{2}}=185.01 \end{array}$$ (2)

The sample size was multiplied by 100/ (1−*NR*), where *NR* is the non-response rate, to account for a 10% non-response rate. As a result, the sample size was multiplied by 100/90 for a 10% non-response rate.

$$\begin{array}{c} n=\frac{185}{0.9}=205.56\approx 206 \end{array}$$ (3)

For the cross-sectional survey, a total of 209 adults were recruited from each of the rural and urban locations, giving a total of 418 participants. A total of 14 participants were involved in the exploratory qualitative study; 10 were from the rural area and four were from the urban area.

### 2.4. Instruments

The non-laboratory-based INTERHEART risk score (NL-IHRS), a validated CVD risk score developed by McGorrian et al. [13], was used to determine the CVD risk status of the participants. The NL-IHRS comprises 11 risk factors, which are age, sex, history of active and passive smoking, diabetes, high blood pressure, family history of heart disease, waist-to-hip ratio, psychosocial factors, diet and physical activity. Each participant's total score on the instrument was used to stratify their CVD risk status as low (score from 0 to 9), moderate (score from 10 to 15) or high risk (score from 16 to 48). The perception of risk of heart disease scale (PRHDS), a 20-item instrument that measures an individual's perception of the probability of developing heart disease [21], was used to investigate participant CVD risk perception. The items on the scale were scored using a 4-point Likert scale ranging from one (strongly disagree) to four (strongly agree). Agreeing with the possibility of having heart disease was denoted as having a positive perception, while declining the possibility of having heart disease was denoted as a negative perception for analysis. The two instruments (NL-IHRS and PRHDS) were translated into Yoruba (an indigenous Nigerian language) with permission from the developers, and the Yoruba versions of the instruments were administered to non-English speakers during the data collection process.

### 2.5. Focus group discussion

Two FGDs were conducted in this study. One group comprised four participants from the urban area (other invited participants could not attend the FGD due to other engagements), while the other group comprised 10 participants from the rural area. Present at each of the FGDs were the researcher, the moderator (who is knowledgeable in qualitative study) and the participants. A focus guide, which is a list of open-ended questions and probes about CVD and perceived CVD risk, was used to guide the discussion. Participants were encouraged to talk freely with an assurance of anonymity, and each discussion was conducted until saturation was achieved. Each FGD was recorded with an audio recorder after consent had been obtained from the participants. The information recorded during the FGDs was transcribed verbatim for analysis.

### 2.6. Statistical analyses

The data from the cross-sectional survey were analyzed by using the descriptive statistics of mean, standard deviation, percentage and frequency. A multivariate logistic regression model was used to investigate the factors that contributed to a high CVD risk status and positive CVD risk perception among the participants. The level of significance *α* was set at 0.05. Content thematic analysis was used to analyze the qualitative data obtained from the exploratory qualitative component of the study.

## 3. Results

### 3.1. Sociodemographic characteristics of participants in the rural and urban locations

A total of 209 adults participated in this study were from the rural area, of whom 117 (56.0%) participants were aged 40 and above, 161 (49.4%) were married and 299 (71.5%) had at least a secondary school education (Table 1). A total of 209 participants were involved in the urban area, of whom 132 (68.4%) participants were aged 40 and above, 165 (78.9%) were married and 199 (95.2%) had at least a secondary school education (Table 1).

**Table 1.** Sociodemographic characteristics of participants in the rural and urban locations.

Variable	Rural n (%)	Urban n (%)	Total (N = 418) n (%)
Sex (n = 412)			
Male	92 (44.0)	95 (46.8)	187 (45.4)
Female	117 (56.0)	108 (53.2)	225 (54.6)
Age (n = 402)			
<40	92 (44.0)	61 (31.6)	153 (38.1)
≥40	117 (56.0)	132 (68.4)	249 (61.9)
Marital status (n = 418)			
Single	17 (8.1)	24 (11.5)	41 (9.8)
Married	161 (77.0)	165 (78.9)	326 (78.0)
Widowed	30 (14.4)	15 (7.2)	45 (10.8)
Others	1 (0.5)	5 (2.4)	6 (1.4)
Level of education (n = 418)
≤Primary	109 (52.2)	10 (4.8)	119 (28.5)
≥Secondary	100 (47.8)	199 (95.2)	299 (71.5)

### 3.2. Cardiovascular disease risk status of participants in the rural and urban locations

Results from this study showed that, of all of the participants in the rural area, 92 (44.0%) had a moderate-to-high risk of CVD, while, in the urban area, 87 (41.6%) participants had a moderate-to-high risk of CVD (Table 2). There was no significant difference (p = 0.115) in the CVD risk status between participants in the rural area and participants in the urban area (Table 2). Participants in the rural and urban areas had a similar risk status.

**Table 2.** Cardiovascular disease risk status of participants in the rural and urban locations.

Variable	Rural (N = 209) n (%)	Urban (N = 209) n (%)	Total (N = 418) n (%)	p-value
Low	117 (56.5)	122 (58.4)	239 (57.2)	0.115
Moderate	78 (37.3)	63 (30.1)	141 (33.7)	
High	14 (6.7)	24 (11.5)	38 (9.1)	

### 3.3. Perceived cardiovascular disease risk of participants in the rural and urban locations

Results from the study showed that, in the rural area, 126 (60.3%) of the participants had a negative perception of CVD risk, while, in the urban area, 109 (52.2%) participants had a positive perception of CVD risk (Table 3). There was a significant difference (p = 0.011) in the distribution of CVD risk perception between participants in the rural area and participants in the urban area (Table 3).

**Table 3.** Perceived cardiovascular disease risk of participants in the rural and urban locations.

Variable	Rural n (%)	Urban n (%)	p-value
Negative perception	126 (60.3)	100 (47.8)	0.011*
Positive perception	83 (39.7)	109 (52.2)	

### 3.4. Sociodemographic variables associated with high cardiovascular disease risk status among all participants

Multiple logistic regression was used to further investigate the sociodemographic variables that are associated with a high CVD risk status among participants, with the strength of the association reported as the odds ratio and confidence interval. Findings showed that males were three times more likely to have a high CVD risk status compared to females in terms of the odds ratio (confidence interval) [3.91 (1.58–9.68)] (Table 4). Also, adults aged 40 years and above were more likely to have a high CVD risk status than younger adults based on the odds ratio (confidence interval) [1.59 (0.63–4.00)] (Table 4). A similar trend was observed for urban-dwelling participants [1.21 (0.33–4.39)]. Participants whose level of education was secondary school and above had lower odds of a high CVD risk status [0.530 (0.12–2.19) (Table 4).

**Table 4.** Odds ratios (ORs) and 95% confidence intervals (CIs) of the multivariate adjustment for the association of sociodemographic variables with cardiovascular disease risk status among participants.

Variable	OR (95% CI)	Level of significance
Sex (male)	3.91 (1.58–9.68)	0.003*
Age (≥40)	1.59 (0.63–4.00)	0.318
Education (≥secondary)	0.530 (0.12–2.19)	0.38
Location (urban)	1.21 (0.33–4.39)	0.76

### 3.5. Sociodemographic variables associated with positive perceived cardiovascular disease risk among all participants

Multiple logistic regression was used to further investigate the sociodemographic variables that are associated with a positive CVD risk perception among participants, with the strength of the association reported as the odds ratio and confidence interval. The results showed that males [0.68 (0.31–1.45)] and those aged 40 years and above [0.74 (0.32–3.03)] had lower odds of a positive CVD risk perception (Table 5), while participants with at least a secondary school education [2.66 (0.61–11.53)] and participants in the urban area [2.62 (0.78–7.08)] had higher odds of a positive perception of CVD risk (Table 5).

**Table 5.** ORs and 95% CIs of the multivariate adjustment for the association of sociodemographic variables with cardiovascular disease risk perception among participants.

Variable	OR (95% CI)	Level of significance
Sex (male)	0.68 (0.31–1.45)	0.31
Age (≥40)	0.74 (0.32–3.03)	0.49
Education (≥secondary)	2.66 (0.61–11.53)	0.19
Location (urban)	2.62 (0.78–8.70)	0.116

### 3.6. Perceived cardiovascular disease risk of participants in the rural and urban locations: Exploratory qualitative aspect of the study

A qualitative study was conducted to further explore CVD risk and risk perception among the participants using FGD. During the discussion, the definition of CVD, possible causes of CVD, problems associated with CVD, prevention of CVD and perception of CVD risk were explored. The FGD included two groups of adults who were purposively selected from the rural and urban areas. To maintain anonymity, participants were assigned numbers and addressed as those numbers during the discourse. Participants were asked questions related to the study's objectives, with probes used as needed. After consent had been obtained from the participants, the information gathered during the discussion was recorded, transcribed verbatim and analyzed using content thematic analysis. Four themes emerged from the qualitative data (Table 6).

**Table 6.** Themes generated from the exploratory qualitative study.

S/N	Themes	Sub-themes
1	Knowledge of cardiovascular disease	Definition of cardiovascular disease Symptoms of cardiovascular disease Possible causes of cardiovascular disease
2	Problems associated with cardiovascular disease	
3	Prevention of cardiovascular disease	
4	Perception of cardiovascular disease risk	Individual perceived risk of cardiovascular disease

#### 3.6.1. Knowledge of cardiovascular disease

Definition of cardiovascular disease. The participants from the rural area were asked to define CVD. Some described it vaguely as “a disease that disturbs”, that it “destroys the body” or that it is “a disease that causes sickness”. Participants in the urban area appeared to have a better understanding of CVD, and a participant in the urban area described it as “a marriage of two words, cardiovascular and disease”. This is shown in the excerpts below:

*“Cardiovascular disease is a disease that destroys the body. It doesn't allow one to have peace of mind. It doesn't allow the body to be as it ought to be”*. (Participant 1, FGD 1, Female, Rural area).

*“Cardiovascular disease... I see it as a marriage of two words, cardiovascular and disease. Cardio has to do with the heart, simply means diseases that are related with the heart, human heart”*. (Participant 2, FGD 2, Male, Urban area).

Symptoms of cardiovascular disease. Participants in the rural area had poor knowledge of the symptoms of CVD and thus mentioned “cough”, “fever” and “headache” as symptoms of CVD, while participants in the urban area had better knowledge and mentioned “chest pain” and “irregular heartbeat” as possible symptoms of CVD. This is shown in the following excerpts:

*“One will have the feeling in the body. The body will not be as it was before. ...The person can be coughing or have fever or headache”*. (Participant 1, FGD 1, Female, Rural area).

Below is an excerpt from the urban area about possible symptoms of CVD:

*“I think it may lead to... the person is prone to chest pain, the person is prone to irregular heartbeat, abnormal heartbeat and can lead to stroke, yeah, as a result of heart disease. You know blood is being pumped from the heart, and when blood is not properly pumped to certain areas, that area will be lacking, deficient in some certain functions and that can lead to chest pain, irregular heartbeat, heart attack and stroke”*. (Participant 1, FGD 2, Male, Urban area).

Possible causes of cardiovascular disease. Participants in the rural area attempted to explain what they thought could be the possible causes of CVD. Some of the possible causes mentioned are “stress”, “anxiety”, “sleeplessness”, “intake of chemicals”, “inappropriate diet” and “hypertension”. Some participants in the urban area mentioned “worry”, “anxiety” and “stress” as possible causes of CVD. This is seen in the excerpts below:

*“My view about it is, hypertension can cause cardiovascular disease because if the blood pressure is high, one will from there have hypertension which is also cardiovascular disease”*. (Participant 3, FGD 1, Female, Rural area).

*“Depression all the time. Like worry, over-anxiety and even too much stress, too much stress can cause it. ...”*. (Participant 4, FGD 2, Male, Urban area).

#### 3.6.2. Problems associated with cardiovascular disease

Participants in the rural area had poor knowledge of CVD; therefore, they repeated the information that they had been previously provided. Participants from the urban area mentioned that problems associated with CVD could be “difficulty in breathing”, “heart attack” and “stroke”, as seen in the excerpts below:

*“Some of the problems associated with it... some have been mentioned like high blood pressure, ah.... Maybe difficulty.... I don't know... difficulty in breathing, as a result of probably excess water in the heart. Hmmm... I don't know maybe this is related with, or....it affects the.... a newborn baby at birth...a situation where there is a hole in the heart ah.... I think those are the major, major problems that could be associated with the heart”*. (Participant 2, FGD 2, Male, Urban area).

#### 3.6.3. Prevention of cardiovascular disease

Participants were asked how CVD may be prevented. Participants in the rural area emphasized the need for the consumption of healthy foods, including proteins, fruits and vegetables. Participants in the urban area demonstrated better knowledge of some CVD preventive practices, as they mentioned “constant medical checkups”, “adherence to medical advice”, “regular exercise”, “healthy diet”, “avoidance of late-night meals” and “avoidance of depression and stress” as ways of preventing CVD. This is shown in the following excerpts:

*“The foods one can eat that will... the foods I think one can eat that can help us... beans, if one eats beans. If one will eat rice, if it is an old person from age 65, it is eggs that are good and fish, not meat. We should eat our rice with vegetables, not just stew or palm oil. So, beans, egg, may God meet our needs, we should be eating it with it. Variety of fruits, whether oranges, that one is close to us... pawpaw... all these things are useful for the body...banana”*. (Participant 4, FGD 1, Male, Rural area).

*“Healthy food is very important because when we begin to eat, junk food, too much junk food, canned food, and builds fat in the body. It thickens the... the... is it veins? The arteries wherein the blood flow and when the arteries are blocked by all these junk, fats, it leads to heart attack and cardiovascular diseases. So, what I'm trying to say is that eating healthy food is very important. By so doing, it will prevent us from heart attack and secondly, regular exercise.”* (Participant 1, FGD 2, Male, Urban area).

#### 3.6.4. Perceived cardiovascular disease risk

Individual perceived risk of cardiovascular disease. Participants in the urban area were asked about their self-perceived risk of having CVD. Most of the participants did not admit their risk of having CVD, even when some of them reported a positive family history of CVD. The following excerpts reveal their thoughts on their perceived risk of CVD:

*“I want to answer this question in two folds, if I should speak as a believer, if I should speak as a Christian who has faith in the Lord and who stays away from some of these ah...who try to work on my diet, I may say, I cannot have it. But if I may speak as a realist because there are people who keep all these rules and regulations, yet they have it. And going by the family history, my mum ah...had high blood pressure and at times, some of these things work in the family”*. (Participant 2, FGD 2, Male, Urban area).

*“I still go back to what I've said. If smoking is taken as part of what can cause cardiovascular disease, I'm exempted. If alcoholism is taken as part of it, I'm exempted. And...exposure to polluted areas as well affects the level of oxygen and if the level of oxygen intake is reduced, it can lead to irregular breathing. So, if one is not exposed to a highly polluted environment, he might not have heart disease”*. (Participant 1, FGD 2, Male, Urban area).

## 4. Discussion

### 4.1. Cardiovascular disease risk status of participants in the rural area

Findings from this study showed that nearly half of the participants in the rural area had moderate-to-high CVD risk. This finding is different from that of Okoro and Jumbo [25], who reported that no participant had a high risk of CVD in a rural area in Bayelsa State, Nigeria. The proportion of individuals with moderate-to-high risk of CVD in the present study is also different from findings from rural communities in Nepal [26] and South India [27], where lower proportions of individuals with moderate-to-high risk of CVD were reported. The higher proportion of individuals with moderate-to-high risk of CVD in the present study could be due to the difference in the type of CVD risk estimation tools used. The presence of CVD risk factors indicates the likelihood of the occurrence of cardiovascular events such as myocardial infarction or stroke in the future [28]. The large proportion of adults with moderate-to-high risk of CVD in the present study is thus of great concern, as this could be a pointer to the looming burden of CVD in the rural community where this study was conducted. CVD prevention programs are needed in rural communities to facilitate the adoption of healthy lifestyle practices to mitigate the rising burden of CVD.

Anecdotal evidence suggests that people living in rural communities in Nigeria have a healthier lifestyle and are less prone to “diseases of affluence”, such as CVD. Findings from the present study, however, seem to contradict this opinion. The study participants from the rural area generally comprised peasant farmers, petty traders and low-income earners, yet, nearly half of them were found to have moderate-to-high CVD risk status. This is attributable to urbanization and the gradual westernization of lifestyle that is taking place in rural communities [29]. There is a need for concerted efforts directed at rural dwellers to improve the awareness of CVD risk factors and promote lifestyle modification. Given the fact that access to healthcare services and the availability of healthcare service providers is generally sub-optimal in rural communities in Nigeria [30], CVD prevention is the best strategy to mitigate the burden of CVD in such areas.

### 4.2. Cardiovascular disease risk status of participants in the urban area

Findings from this study showed that nearly half of the participants in the urban area had moderate-to-high risk of CVD. This finding is consistent with that of previous studies from urban areas in Brazil [31], Cameroon [32] and Indonesia [33], where the proportion of individuals with moderate-to-high risk of CVD was about half of the total sample of participants. Adedoyin et al. [34] in a study among university staff in South West, Nigeria, found that less than 10% of the participants had a high risk of CVD. This proportion is lower than that which was found in the present study. This difference is attributable to the rising prevalence of CVD and CVD risk factors [10], since the previous study by Adedoyin et al. [34] was conducted 4 years earlier. The difference in the CVD risk estimation tools utilized is another possible reason for this difference. Urbanization has been identified as a factor that increases the risk of CVD [35]; therefore, as urbanization continues, the risk of CVD is projected to increase among urban-dwelling adults. There is a need for the development of individual- and population-based CVD prevention programs targeted at high-risk individuals and the entire sub-population of adults in the urban area to curb the burden of CVD.

CVD risk status estimation and stratification as low, moderate or high risk, as done in this study, is one of the most important steps toward reducing the incidence of CVD [27]. Evidence has shown that people with moderate-to-high CVD risk status are at risk of cardiovascular events in the future [36]. Furthermore, individuals with a high-risk status are twice as likely to develop CVD as compared to those with a low-risk status [34]. The proportion of participants with moderate and high CVD risk status in this study reveals the looming burden of CVD among adults both in the urban and rural areas in Oyo State. To mitigate this, comprehensive prevention and management strategies tailored to each of the rural and urban communities are required.

### 4.3. Difference between cardiovascular risk status of adults in rural and urban areas

Findings from this study showed that participants in the urban area had a slightly larger proportion of individuals in the high-risk category as compared to participants in the rural area. Previous studies have reported a higher proportion of high-risk individuals in urban areas. Gong et al. [37] reported that, as compared with rural areas, urban areas had higher incidence and mortality rates for coronary heart disease in China. Maharani et al. [33] also reported that the proportion of participants with high CVD risk was greater in urban areas than in rural areas among Indonesians. Another study by Forero et al. [38] among patients with co-morbid bipolar disorder in urban and rural areas in Colombia found that rural patients with the disease were more likely to have a more favorable CVD risk status than urban patients with the disease. It is commonly believed that CVD risk is higher among urban-dwelling individuals than among their rural-dwelling counterparts [39]; this was also confirmed in this study. However, the difference found between the urban and rural areas in the present study was small compared to what was observed in other countries.

In the present study, there was no significant difference in CVD risk status between participants living in the rural area and those living in the urban area. Participants in the rural and urban areas generally had similar proportions of individuals in the “low”, “moderate” and “high” risk categories. This can be explained by the high prevalence of unhealthy lifestyle practices, such as poor dietary patterns among rural- and urban-dwelling participants. There is an ongoing westernization of lifestyle in rural areas, whereby rural dwellers now consume more processed foods and are less physically active than what was obtainable some decades ago. Yusuf et al. [40] from the global PURE cohort from high-income countries, reported similar INTERHEART risk scores between populations in rural and urban areas. Findings from the study also indicated that the rural community had a larger proportion of participants with only primary education than the urban area. Low educational levels could predispose individuals to unhealthy lifestyle choices due to poor knowledge of the implication of such choices. A low educational level has been associated with a greater predisposition to the development of CVD [41]. More attention should therefore be given to the rural areas by healthcare stakeholders and policymakers to improve the awareness of CVD risk factors and develop CVD prevention programs for rural-dwelling adults, taking into account the cultural and socioeconomic contexts. Urban-dwelling adults should also be targeted for effective CVD prevention programs to curb the rising burden of CVD.

### 4.4. Cardiovascular disease risk perception of participants in the rural area

Findings from this study showed that the majority of the participants in the rural area had a negative perception of their risk for CVD. This finding is similar to that of Odunaiya et al. [42], who performed a study among university students in Nigeria where the majority of the participants were found to have a negative perception of CVD risk. Mohd-Azahar et al. [43], in a prospective study among rural Malaysians with hypertension, found that participants underestimated their risk for future cardiovascular events. Meanwhile, hypertension is the leading risk factor for CVD [44]. Another study in Nigeria by Umuerri [45] found that, although participants perceived heart disease to be a serious condition, the majority of them were not concerned about their risk of getting the disease. The negative perception of CVD reported by more than half of the rural-dwelling participants in this study is attributable to the religious and cultural beliefs held in Nigeria, where most people tend to decry “negative confessions” about their health and, thus, fail to admit their possibility of having a disease even when they are evidently at risk.

Research suggests that poor perception of CVD risk affects the adoption of healthy lifestyle habits [46], which is the cornerstone of CVD prevention [47]. The exploratory qualitative study provided more insight into the participants' perceptions of CVD and its risk factors. Qualitative studies on CVD risk perception are sparse in Nigeria; however, results from the qualitative aspect of the present study showed that some of the participants, particularly in the rural area, did not have basic knowledge of CVD; meanwhile, knowledge is a major factor that influences perception. Some of the participants described CVD vaguely as a disease that destroys the body and that can cause premature death. Concerning the symptoms, participants in the rural area mentioned headaches, coughing and joint pains as possible symptoms, while one of the participants in the urban area was able to identify chest pain and an irregular heartbeat as possible symptoms of CVD. Findings from this study reveal the need for intensive health education and CVD awareness interventions targeted at rural populations to enhance the proper perception of CVD and thus aid lifestyle modification and behavioral changes among rural dwellers. This must, however, be done within the religious, socioeconomic and cultural contexts of the rural-dwelling adults in mind to enhance effectiveness.

In the present study, participants in the rural area particularly emphasized stress, excessive thinking and inadequate sleep, especially as it relates to home, work and socioeconomic demands, as possible causes of CVD. This is attributable to the low socioeconomic status and the high level of poverty among the rural dwellers, as the majority of them were peasant farmers and petty traders. Wekesah et al. [48] previously reported that stress was a commonly identified CVD risk factor among adults in Kenya. Women were especially singled out as those likely to develop CVD as a result of stress and worry.

### 4.5. Cardiovascular disease risk perception of participants in the urban area

Findings from this study showed that, among the participants in the urban area, the majority had a positive perception of CVD risk. This finding is in tandem with that of Roos et al. [49] in South Africa, where a large proportion of the participants reported a positive perception of CVD risk because of their poor health behavior. Also, Abdela et al. [50], in a study among undergraduate students in Ethiopia, also reported that most participants showed a positive perception toward CVD risk factors. The large proportion of individuals with a positive perception of CVD risk in the present study could be a result of better education. Findings from the qualitative component of the study revealed that participants in the urban area were able to give more appropriate definitions of CVD. They also provided a better description of the symptoms of CVD, stating symptoms such as chest pain, irregular heartbeat and breathing difficulty. This could be because participants in the urban area are generally more educated. Virtually all participants in the urban area had at least a secondary school education. This could foster better knowledge of CVD and its risk factors among urban dwellers. Also, people living in urban areas tend to have better access to information on CVD risk through various media outlets, and they have better access to medical advice. In the present study, we found that some participants in the urban area also had a negative perception of CVD risk. More health promotion interventions are required among urban-dwelling adults to improve the proper perception of CVD and promote the adoption of a healthy lifestyle in order to reduce the burden of CVD.

The participants in the urban area in this study identified difficulty in breathing, chest pain, abnormal heartbeats, blockage of arteries, stroke and a heart attack as problems that could be associated with CVD. Participants in the urban area demonstrated good knowledge of the problems that could occur as a result of CVD. This is useful in the promotion of healthy lifestyle practices among this sub-population. Participants in the urban area provided relevant information on ways by which CVD may be prevented, with particular emphasis on healthy diet and exercise. This could be a result of increasing awareness of the importance of a healthy diet and physical activity in the prevention of non-communicable diseases in urban areas.

Findings from the qualitative aspect of this study on the perceived risk of CVD revealed that most participants did not perceive themselves as being at risk of having CVD, even when some of them admitted a positive family history of the disease. Participants in the present study did not take responsibility for having certain unhealthy lifestyle practices that could put them at risk of CVD; they instead confessed their faith in God against the disease. This could be a result of the religiosity of most Nigerians and the cultural belief that “what you say is what you have”. This agrees with the report of Surka et al. [51], who also submitted that personal, cultural and religious beliefs influenced how risk was perceived among adults in South Africa. Participants in the present study preferred to stay in denial than admit their risk of having CVD in the future. Some participants also thought that, since they do not drink nor smoke, they are not at risk of having CVD. However, this opinion is not right, as CVD often occurs as a result of a conglomeration of risk factors, both modifiable and non-modifiable. Individual perception of CVD risk can greatly influence perceived susceptibility and health behavior [22]. There is therefore a need for CVD awareness campaigns, health education and promotion for adults in urban and rural areas to facilitate proper perception of CVD and ultimately promote CVD prevention practices.

### 4.6. Sociodemographic factors associated with cardiovascular disease risk status among all participants

A multiple logistic regression model was used to further investigate the sociodemographic variables associated with a high CVD risk status among the participants. It was necessary to identify the groups at higher risk of CVD among the participants to promote the design of appropriate intervention measures for each group. Multiple logistic regression in this study showed that males were three times more likely to develop CVD than females. This is in keeping with the findings of Alikor and Emem-Chioma [29], who reported a higher clustering of risk factors among the male gender. This higher risk in men compared with women may be because some of the CVD risk factors evaluated in this study were in higher proportions for males than females. Furthermore, whereas men were found to have a higher absolute risk of CVD, women were found to have a higher relative risk of CVD morbidity and mortality. Men appear to be at a higher risk of having CVD than premenopausal women, although the risk is about the same after menopause [52]. Findings from the present study also revealed that being an adult of age 40 and above is associated with a high CVD risk status. This is consistent with the findings of Okoro and Jumbo [25] and Alikor and Emem-Chioma [29], who also found an association between age and CVD risk. Advancing age has been established as an independent, non-modifiable risk factor for CVD [53]. The risk of having a cerebrovascular disease, for example, doubles every decade after the age of 55 [54]. A possible reason is that aging is associated with a progressive decline in numerous anatomical structures and physiological processes. Its remarkable effect on the endothelium of the arterial system leads to a thickening of the arterial walls, increasing the risk of the occurrence of atherosclerosis [55]. The atherosclerotic changes that occur with aging result in various cardiovascular pathologies, such as left ventricular hypertrophy, chronic heart failure and atrial fibrillation [56].

Furthermore, findings from this study showed that adults with an educational level below secondary are more likely to have CVD. This finding is in agreement with a previous study by Degano et al. [41] that reported an inverse relationship between educational attainment and the incidence of CVD. Lifestyle choices and health behavior are often dependent on the level of cognition, problem-solving skills and ability to make informed choices, which may be lacking in people with a low level of education [57]. There is therefore a need for CVD awareness programs and health education to be targeted at individuals at various levels of education to obtain meaningful results. The present study also showed that living in an urban area was associated with high CVD risk status. This is in tandem with the findings of Maharani et al. [33], who found that respondents in semi-urban and urban areas had higher odds of having high cardiovascular risk than those in rural areas. The association between high CVD risk and living in an urban area could be explained by the westernization of the lifestyle of people living in the urban area, with a tendency toward unhealthy diets and low physical activity levels. More attention should be given to adults living in urban areas to promote healthy lifestyle choices, thus reducing the incidence of CVD.

### 4.7. Sociodemographic variables associated with cardiovascular disease risk perception among all participants

The results of multiple logistic regression showed that being female was a factor that contributed to a positive perception of CVD risk. This is consistent with the findings of Muhihi et al. [58], who reported that women perceived themselves to be more at risk of CVD than men. The present study also revealed that those below age 40 had better CVD risk perception. This is in tandem with the findings of Obembe et al. [59], who reported that being below age 40 was a significant predictor of the knowledge of CVDs, and that knowledge of CVD informs perception. Participants with at least a secondary school education were two times more likely to have a positive perception of CVD risk than those without it. This agrees with the findings of Aminde et al. [60] in Cameroon, where a high level of education also predicted moderate-to-good knowledge of CVD. This is not surprising because educated people have better access to information and greater cognitive capacity to comprehend the information on CVD and other health concerns often delivered on various media channels. The proportion of participants in the urban area with a positive perception of CVD risk was more than twice that of those in the rural area. This could also be a result of better access to information on CVD risk and the ability to make informed decisions. Although knowledge and perception are separate constructs, good knowledge promotes proper perception. Findings from this study have shown the need for an improvement of CVD knowledge and awareness to enhance the proper perception of CVD risk among rural and urban dwellers. An understanding of the perception of CVD risk in various sub-populations is crucial to inform the design and implementation of context-appropriate interventions to mitigate the rising burden of CVD. This will, in turn, promote healthy lifestyle changes to reduce the burden of CVD among adults in urban and rural areas in Oyo state, Nigeria.

This study was conducted in the southwest zone of Nigeria; therefore, its findings cannot be generalized to the whole of Nigeria. There is a need for a nationwide study to determine the perceived CVD risk and risk status of adults in other parts of the country. Nevertheless, this study provides useful information on the perceived risk and CVD risk status of adults in rural and urban areas in Oyo State. This information may serve as a basis for the development of CVD prevention programs for adults in urban and rural areas in Oyo State, and it can stimulate further research on CVD prevention in Nigeria.

## 5. Conclusions

There was a large proportion of adults with a moderate-to-high risk of CVD among the participants in the urban and rural communities in Oyo State. Most adults, particularly in the rural area, tended to have a negative perception of CVD risk. Being male, being age 40 or above, having an educational status below secondary and living in an urban area were important factors associated with a high CVD risk status. Being female, being less than 40 years old, having at least a secondary school education and living in an urban area were the important factors associated with a positive CVD risk perception in this study. There is an urgent need for health education and CVD awareness campaigns among adults living in urban and rural communities in Nigeria. Interventions should be context-specific and should consider the religious and sociocultural beliefs of adults, especially in rural areas.
